# A Comparative Effect of Different Herbal Products on Lipid Metabolism and Hepatic Tissue: An Experimental Study on a Rat Model

**DOI:** 10.7759/cureus.73799

**Published:** 2024-11-16

**Authors:** Saman H Mohammed, Jamal K Shakor, Mohsin Salih, Kaniaw Khafar, Halgord M Ali, Hardi R Baqi, Dyar H Karim, Shagul J Muhammd, Chrakhan J Khdhir, Chro Raouf

**Affiliations:** 1 Nursing, Darbandikhan Technical Institute, Sulaimani Polytechnic University, Sulaymaniyah, IRQ; 2 Nursing, College of Health and Medical Technology, Sulaimani Polytechnic University, Sulaymaniyah, IRQ; 3 Nursing, Technical College of Applied Sciences, Sulaimani Polytechnic University, Sulaymaniyah, IRQ; 4 Nursing, Technical College of Applied Sciences, Halabja Research Center, Sulaimani Polytechnic University, Sulaymaniyah, IRQ; 5 Medical Laboratory Technology, Shaqlawa Technical College, Erbil Polytechnic University, Erbil, IRQ; 6 Biology, College of Science, University of Sulaimani, Sulaymaniyah, IRQ; 7 Animal Science, College of Agricultural Engineering Sciences, University of Sulaimani, Sulaymaniyah, IRQ; 8 College of Nursing, University of Human Development, Sulaymaniyah, IRQ; 9 Medical Laboratory of Science, College of Health Sciences, University of Human Development, Kurdistan Regional Government, Sulaymaniyah, IRQ

**Keywords:** dyslipidemia, japanese powder tea, lipid profile, liver abnormalities, shahana tea, slimming pill

## Abstract

Background

Dysregulation of lipid metabolism can lead to conditions such as hyperlipidemia, obesity, cardiovascular diseases, and hepatic steatosis. A high-fat diet (HFD) results in dysregulation of lipid metabolism and may primarily convert liver tissue to develop inflammation and fibrosis. Slimming pills, Japanese powder tea, and Shahana tea are common green teas that commercials have used for hyperlipidemia, obesity, and liver protection. The aim of this study was to investigate the effect of these three teas on dyslipidemia and liver in a rat model.

Method

This is an experimental study carried out on 20 adult male albino rats of about 240 g and 12 weeks old. The rats were randomly divided into five groups: Group 1: fed the standard pellet diet for four weeks; Group 2: fed with the HFD for four weeks; Group 3: fed with the HFD for the first four weeks and received Shahana tea (1.5  g/kg body weight (BW)); Group 4: fed with the HFD for the first four weeks and received Japanese powder tea (1.5  g/kg BW); and Group 5: fed with the HFD for the first four weeks and received slimming pill (0.6  g/kg BW). Blood samples were collected to measure the lipid profile in the rats. The rats were scarified under anesthesia, and liver tissue was collected for histopathological testing.

Result

HFD could significantly induce dyslipidemia and liver pathological disorders in model rats. Slimming pills could significantly improve total cholesterol (TC), triglycerides (TG), high-density lipoprotein (HDL), and low-density lipoprotein (LDL) compared to Japanese powder tea and Shahana tea. In comparison to the Shahana tea, Japanese powder tea had a significant outcome on LDL but not on other lipid profiles. Slimming pills and Shahana tea could preserve the normal histological features of the liver. The central vein (CV) and sinusoidal (SN) Kupffer cells significantly remained normal compared to model rats.

Conclusion

Slimming pills and Shahana tea have significant positive effects on lipid metabolism regulation, dyslipidemia, and preserving the liver from injury and fat accumulation. The effects of the two products are mostly concerned with their main components, such as L-carnitine and *Cassia angustifolia*.

## Introduction

Dyslipidemia contributes to the major portion of deaths attributed to cardiovascular diseases, metabolic syndrome, and fatty liver diseases. In the last three decades, cardiovascular-related deaths due to high consumption of low-density lipoprotein cholesterol (LDL-C) and non-HDL-C have increased to double [1]. High-fat-containing foods, such as saturated fat, induce lipid metabolic dysregulation, obesity and hyperlipidemia, atherosclerosis, liver fibrosis, and other pathophysiological abnormalities that develop into serious health problems and diseases [1-3]. Hyperlipidemia is associated with a three-fold higher risk of heart attack compared to people with normal cholesterol levels [2].

Regular lipid metabolism is crucial for producing and storing energy, constructing cell membranes, cellular function, and overall metabolic health. Lipid metabolism encompasses many biochemical and physiological processes that primarily involve the absorption of cholesterol and triglycerides (TG) from enterocytes of the small intestine, converting them to chylomicrons in the bloodstream. After a few lipolysis, the remaining chylomicron is converted by the liver to very low-density lipoprotein (VLDL) and released to the bloodstream, which is converted to IDL and to low-density lipoprotein (LDL) in the bloodstream once more. After that, LDL is mostly absorbed by muscle and adipose tissue, and they are stored as cholesterol or metabolized by the process of lipolysis. Meanwhile, HDL mainly mediates or takes the role of transporting the cholesterol from the peripherals cells to the bloodstream and liver. The main portion of plasma cholesterol is synthesized from the liver indigenously, with less than one-fifth deriving from the food from the intestinal. At the same time, triglyceride is mainly derived exogenously from dietary food in the intestine. Plasma LDL is produced as the result of the metabolism of cholesterol and TG in the liver. LDL could be converted and stored to cholesterol in a muscle and deposed tissue [4].

The liver plays a leading role in body energy regulation and metabolism; it contributes to the digestion and metabolism of carbohydrates, amino acids, and lipids. Increased consumption of a lipid-source diet would significantly affect the liver’s pathophysiology and its functions. A study has revealed that a high-fat diet (HFD) leads to increases in TG levels and lipid droplet formation in hepatocytes, which in turn leads to impaired autophagy and cellular apoptosis [5]. Accumulation of lipids in the liver contributes to distorting the liver parenchymal and non-parenchymal cells and induces the dysfunction of hepatocytes and polarization of Kupffer cells [6]. Hepatic steatosis is one of the pathological features that occurs as the result of the accumulation of fat in the liver, which mainly develops into inflammation and fibrosis [7].

Today, many herbal teas have been recommended to decrease obesity and HFD risk factors and dyslipidemia complications such as cardiovascular diseases. Green tea, such as Japanese powder tea, is one of the most common herbal teas that gained more attention for producing positive physiological effects on health, improving the serum lipid profile, and possessing antioxidant, anti-cancer, and anti-inflammatory effects. Green tea, due to its polyphenolic component, has been used for the therapy of dyslipidemia and to ameliorate HFD risks such as obesity, atherosclerosis, and liver pathological disorders [6,8,9].

Using various green teas as therapy for weight reduction, prevention of liver and metabolic syndromes, and treatment of dyslipidemia requires further studies for their pharmacological benefits and safety depending on their components and compositions. Thus, the aim of this study was to investigate the effect of different teas, such as slimming pills, Japanese powder tea, and Shahana tea, on dyslipidemia in a rat model subjected to HFD.

## Materials and methods

Method and subject

This is an experimental study in which 20 male adult albino rats of about 240 ± 10 g body weight (BW) and 12 weeks old were used. The animals were housed in plastic cages bedded with wooden chips and bred in an animal house that belonged to the Biology Department, College of Science and Education, University of Sulaimani. This study has been passed through and approved by the ethical committee of Sulaimani Polytechnic University. During the experimental period, four animals were kept in each cage, and they were housed under standard laboratory conditions (12:12 light/dark at 23 ± 2°C). One rat cage was recruited as a control, and the other four cages were selected as experimental groups.

Rat’s diet

The rats were fed either the normal diet or HFD for four weeks to induce hyperlipidemia and liver pathological distortion. The control rats were fed with a normal standard diet, and the experimental rats were fed with HFD. The HFD was prepared by mixing the control diet with 1.5% cholesterol, 20% palm oil, and 0.25% cholic acid.

Study design

The rats were randomly divided into five experimental groups as follows: Group 1 (control group): fed the standard pellet diet for four weeks; Group 2 (model group): fed with the HFD for four weeks; Group 3: fed with the HFD for the first four weeks and received Shahana tea (1.5  g/kg BW); Group 4: fed with the HFD for the first four weeks and received Japanese powder tea (1.5  g/kg BW); Group 5 fed with the HFD for the first four weeks and received slimming pill (0.6  g/kg BW). 

Material composition

The three herbal teas are accessible in the Kurdistan Market and online market. People usually consume them for weight loss and better health. Slimming pills were purchased online, and according to their brochure, the gradient of one capsule is made from yerba mate tea (150 mg) and L-carnitine (150 mg), *Garcinia cambogia* (75 mg), and green tea extract (50 mg) with vitamins B3 (16 mg) and B6 (1.3 mg). It is commercially used for weight loss since the component has anti-obesity bioactivity.

This Shahana tea was purchased from Sarchnar Supermarket in Sulaimani, Iraq, and according to their brochure, it is made from six extracted herbal ingredients. The percentage of each composition has been indicated based on its proportion in the tea's weight. The Shahana tea contains many components such as *Cassia angustifolia* (40%), *Juniperus communis* (30%), *Ocimum basilicum* (25%), sage leaves (10%), mint leaves (10%), and *Ceratonia* (5%).

Japanese powder tea was purchased online; it is composed of a different gradient of polyphenols and other green tea components. 

Collection and preparation of blood samples

At the culmination of the study period, the rats were fasted overnight and subsequently anesthetized via an intraperitoneal injection of a ketamine hydrochloride (50 mg/kg BW) and xylazine (5 mg/kg BW) mixture. Blood samples were then drawn through a cardiac puncture into gel tubes, which were centrifuged at 3000 rpm for 15 minutes. The resulting serum was analyzed for biochemical parameters.

Histopathology test

Histopathological tests were done on all rats’ liver tissue samples to observe histopathological abnormalities. The collected liver tissue was cleaned with 1% cold saline and then preserved with 10% formalin. The samples are processed and fixed in paraffin blocks and cut into 3 μm slices by microtome. The fixed slices were stained with hematoxylin and eosin (H&E) stain and immobilized on glass slides. The examination was done using a binocular microscope, and light micrographs of the fixed organ were obtained. Histopathological abnormalities were observed from glass slides and have been shown in the figures.

Estimation of serum biochemical

After blood sample collection, the serum biochemical parameters, including serum cholesterol, serum TG, serum high-density lipoprotein, and serum LDL, were quantified using an automated biochemistry analyzer (Cobas E 411, Roche Diagnostics GmbH, Mannheim, Germany). All lipid profile indicators were measured by using mg/dL.

Statistical analysis

The IBM SPSS Statistics for Windows (IBM Corp., Version 26, Armonk, NY) application was used to analyze the data. The Kruskal-Wallis test was employed to test the null hypothesis for the distribution of the lipid profile measures’ values among the different rat groups. Additionally, the pairwise comparisons of groups test was used to test the significant differences in lipid profile value between each pair group. The significance level is calculated at the 0.05 level. Histopathological results of liver tissue have been demonstrated in figures and images.

## Results

The independent-samples Kruskal-Wallis test has demonstrated that the distribution of all lipid profiles among various groups of rats was significantly different (<0.05), and the values of total cholesterol (TC), TG, HDL, and LDL were significantly different among the rats groups, control, model, slimming pill, Japanese powder tea, and Shahana tea groups (Table 1).

**Table 1:** Independent-samples Kruskal-Wallis test for lipid profile among rats group Asymptotic significances are displayed. The significance level is 0.050. HDL, high-density lipoprotein; LDL, low-density lipoprotein; TC, total cholesterol; TG, triglycerides

	Null hypothesis	Significance level	Decision
1	The distribution of TC is the same across all rat groups.	0.008	Reject the null hypothesis.
2	The distribution of TG is the same across all rat groups.	0.015	Reject the null hypothesis.
3	The distribution of HDL is the same across all rat groups.	0.008	Reject the null hypothesis.
4	The distribution of LDL is the same across all rat groups.	0.007	Reject the null hypothesis.

**Figure 1:**
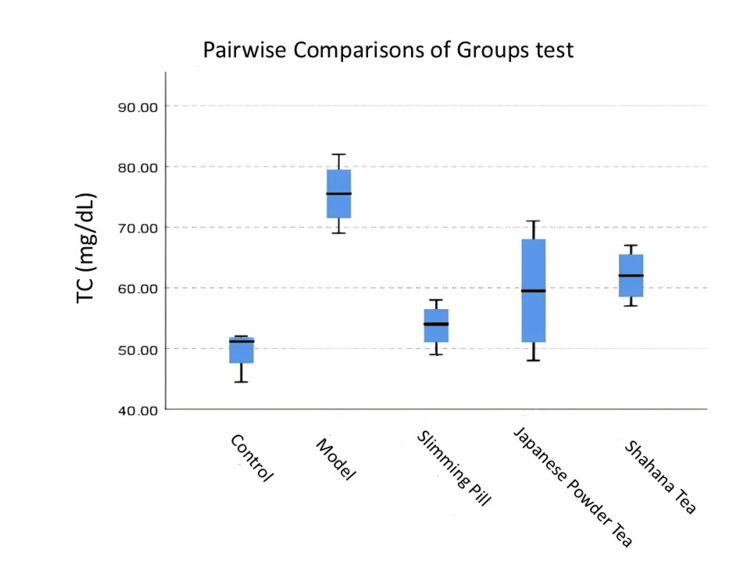
Pairwise comparisons of groups test for TC in each pair groups The distribution of TC among the different groups. Pairwise comparisons of groups test: p-value for control-Shahana tea was 0.027, control-model was 0.000, and slimming pill-model was 0.017. TC, total cholesterol

Figure 1 shows that the rats in the Shahana tea and model groups had significantly high TC compared to the rats in the control group (<0.05). The TC level in the capsule was significantly low compared with the rats in the model group (<0.05). The pairwise comparisons of the groups test showed no significant differences between Japanese powder tea-Shahana tea, Japanese powder tea-model, Japanese powder-slimming pill, and royal-model groups in terms of TC value (>0.05). 

**Figure 2:**
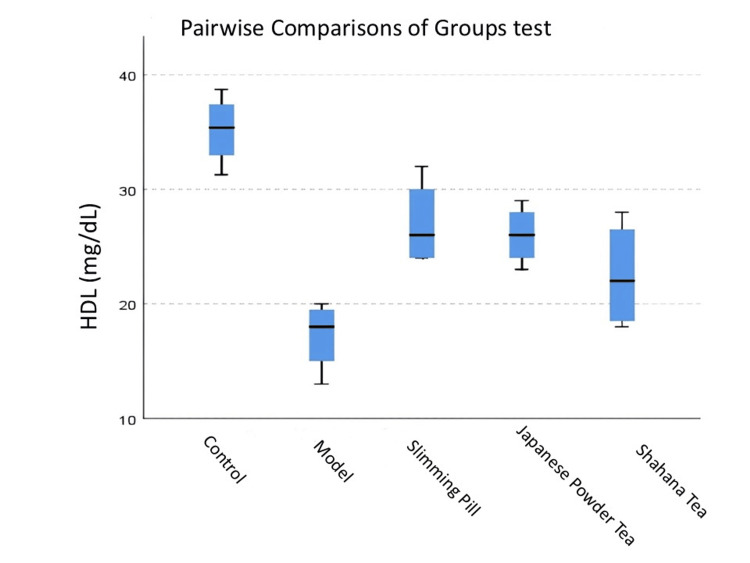
Pairwise comparisons of groups test for HDL in each pair groups The distribution of HDL among different groups. Pairwise comparisons of groups test: p-value for control-Shahana tea was 0.042, for control-model was 0.000, and for slimming pill-model was 0.013. HDL, high-density lipoprotein

Figure 2 shows that the rats in the model groups had a high TG level compared to the rats in the control group (<0.05). TG level only in the slimming pill group was significantly decreased when compared with the model group (<0.05). The pairwise comparisons of groups showed no significant differences in TG level between the control-slimming pill, control-Japanese powder tea group, and control-Shahana tea group, Japanese powder tea-Shahana tea, Japanese powder tea-model, and Shahana tea-model groups (>0.05).

**Figure 3:**
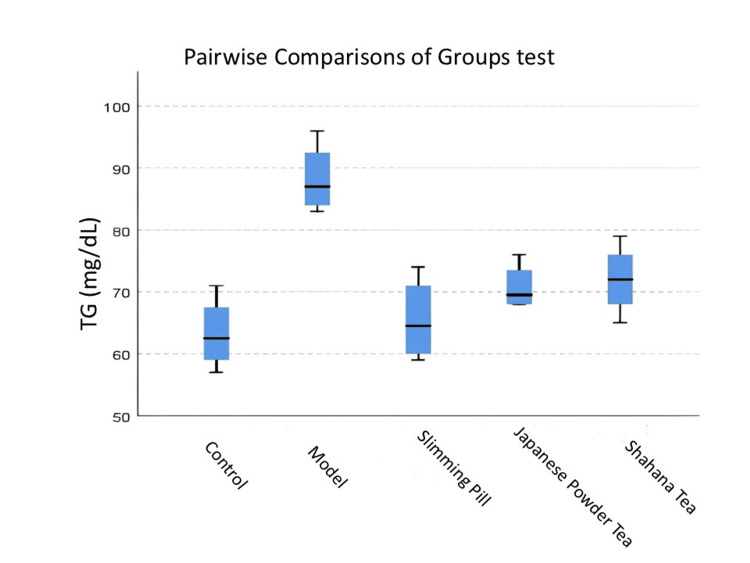
Pairwise comparisons of groups test for TG in each pair groups The distribution of TG among different groups. Pairwise comparisons of groups test: p-value for control-model was 0.001, and for slimming pill-model was 0.005. TG, triglycerides

Figure 3 shows that the rats in the Shahana tea and model groups had significantly low HDL compared to the control group (<0.05). HDL in the slimming pill group was significantly high compared to the model group (<0.05). In contrast, the pairwise comparisons of groups demonstrated no significant differences in HDL levels between the control-slimming pill, Japanese powder tea-Shahana tea, Japanese powder tea-model, Shahana tea-model, and control-Japanese powder tea groups (>0.05).

**Figure 4:**
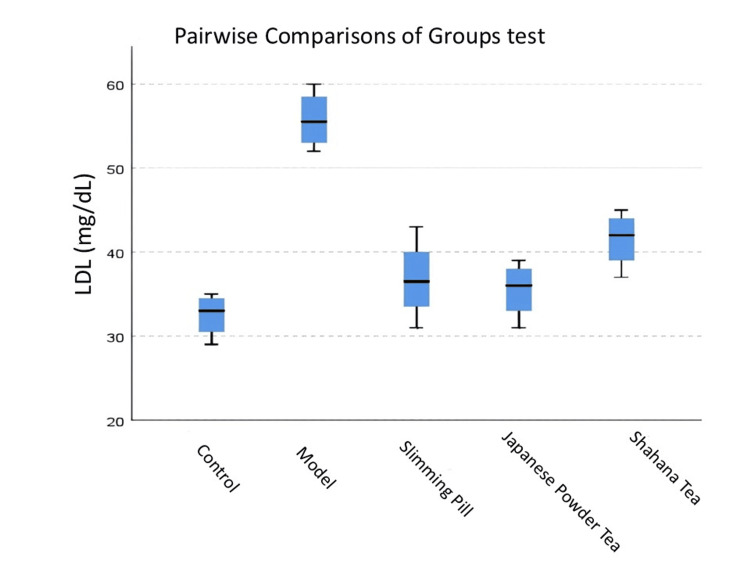
Pairwise comparisons of groups test for LDL in each pair groups The distribution of LDL among different groups. Pairwise comparisons of groups test: p-value for control-Shahana tea was 0.027, for control-model was 0.001, for slimming pill-model was 0.010, and for Japanese powder tea-model was 0.019. LDL, low-density lipoprotein

Figure 4 shows the significantly high LDL level in the Shahana tea and model groups compared to the control group (<0.05). LDL level in the slimming pill and Japanese powder tea group was significantly low compared to the model group (<0.05), while the pairwise comparisons of groups demonstrated no significant differences in LDL level between the control-slimming pill, Japanese powder tea-Shahana tea, Shahana tea-model, and control-Japanese powder tea groups (>0.05).

## Discussion

This study was designed to investigate the effect of different herbal teas, such as slimming pills, Japanese powder tea, and Shahana tea, on lipid profile and liver tissue in a rat model. In this study, model rats were subjected to HFD, which induced a significant increase in TC, TG, and LDL levels in the rat model compared to the control group. The independent-samples Kruskal-Wallis test has demonstrated that the effect of different herbal products on lipid profile significantly varied; particularly, slimming pills could significantly improve the lipid profile values compared to Shahana and Japanese powder teas (<0.05). According to the results of the pairwise comparisons of groups test, the slimming pill had a better outcome on TC, TG, HDL, and LDL compared to Shahana and Japanese powder teas. The main components of slimming pills are L-carnitine and Yerba Mate, and their effects on the regulation of fatty acid metabolism are significant. Both L-carnitine and yerba mate could regulate lipid metabolism by various mechanisms and pathways. L-carnitine carries the acyl groups across cell membranes; L-carnitine transports the long-chain fatty acyl CoAs into the mitochondria for degradation by β-oxidation; long fatty acids would be transferred into mitochondria [10,11]. A study has indicated that supplementing yerba mate for 12 weeks would decrease BW and visceral and abdominal fat. Yerba mate contains hydroxycinnamic acids, caffeine, theobromine, and theophylline, and it can decrease levels of cholesterol, TG, and LDL in the blood [12,13].

In comparison to the Shahana tea, Japanese powder tea produced a significant improvement in LDL only. Concerning other lipids, TC, TG, and HDL, matcha tea was not significantly more effective than Shahana tea. However, concerning the model rat, Japanese powder tea, which is composed of caffeine, catechins, and theanine, could improve lipid profile remarkably. A study indicated that Japanese powder tea in HFD rats could significantly reduce serum TC, TG, and LDL and increase HDL serum [14,15]. The mechanisms of catechin activity include antioxidants, inhibition of redox-sensitive transcription factors, and inhibition of pro-oxidant enzymes [16]. Catechin takes a significant role in lipid metabolism regulation by involving two enzymes, increasing AMP-dependent protein kinase activity and decreasing acetyl-coenzyme A (CoA) carboxylase (ACC), which has a role in metabolism in liver and subcutaneous adipose tissue by fatty acid composition [17]. The *Cassia angustifolia *component of Shahana tea has improved the lipid profile in tetrachloride-induced rats; *Cassia angustifolia* can decrease the cholesterol absorption in the intestinal and catabolism of cholesterol in the liver to form bile duct [18]. However, in this study, Shahana tea could not significantly improve serum lipid profile.

**Figure 5:**
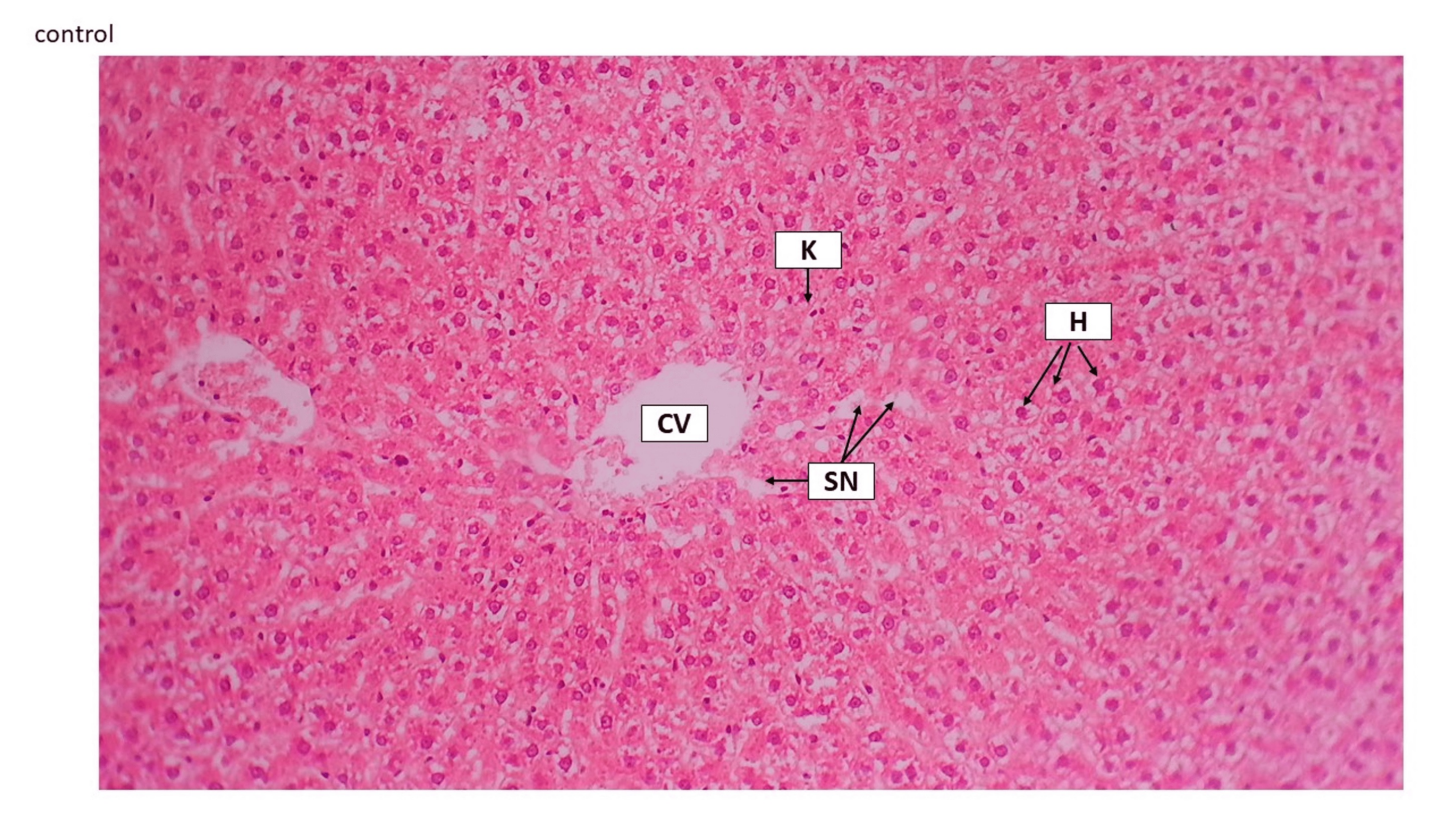
Control liver tissue The control group revealed liver histology, central vein (CV), sinusoids (SN), hepatocytes (H), and Kupffer cells can be seen (K). H&E staining 10×. H&E, hematoxylin and eosin

**Figure 6:**
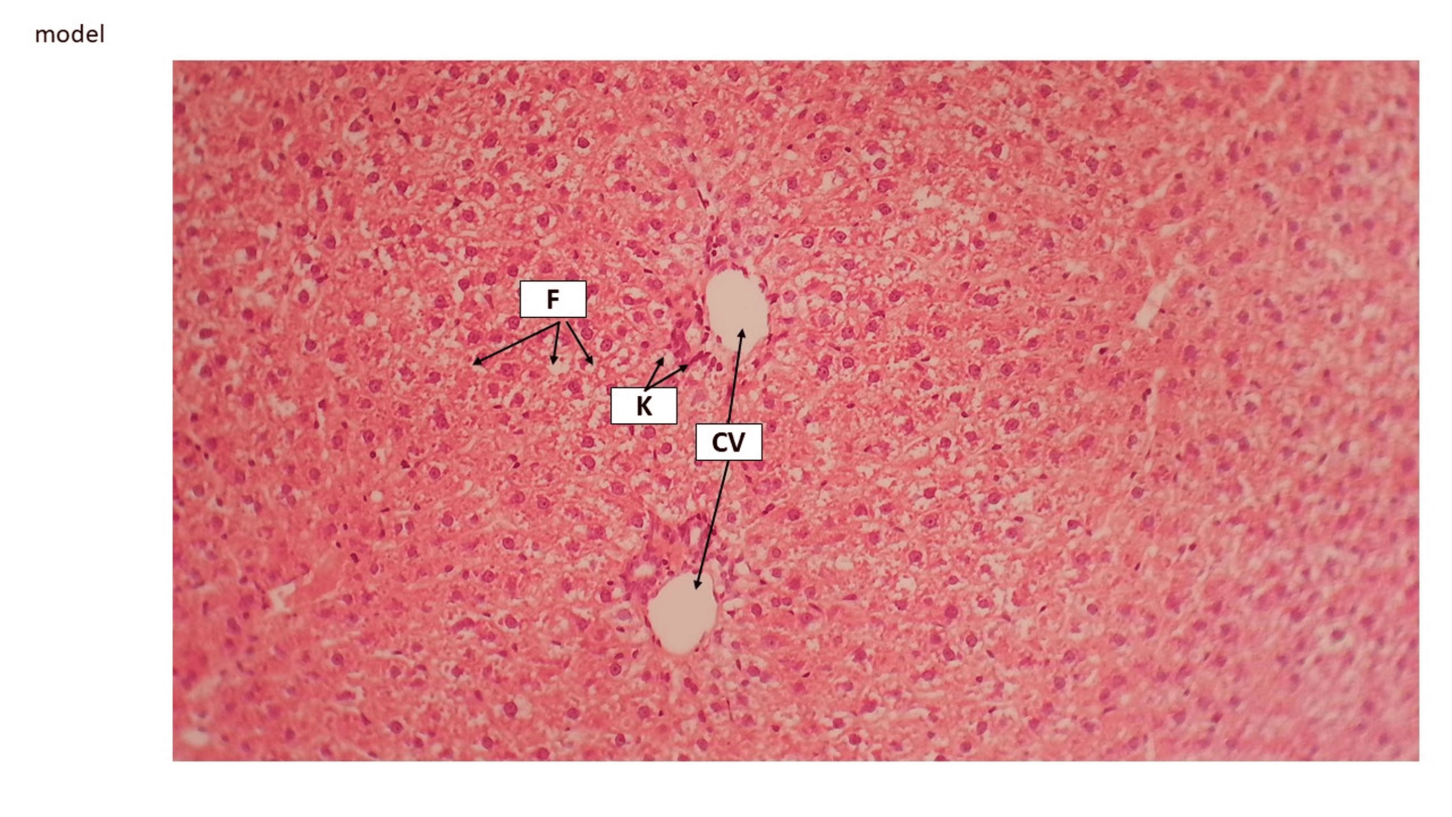
Model liver tissue The model group revealed a central vein (CV). Sinusoids disappeared due to hepatocytes swelling by fatty change (F). There is hyperplasia of Kupffer cells (K). H&E staining 10×. H&E, hematoxylin and eosin

In the current study, HFD (1.5% cholesterol, 20% palm oil, and 0.25% cholic acid) led to histopathological distortion in liver tissue. The hepatic tissue of the model rat indicated that hepatocytes were swollen and occupied by high fatty acids, which led to sinusoids being vanished and hyperplasia of Kupffer cells being realized (Figure 5 and Figure 6).

**Figure 7:**
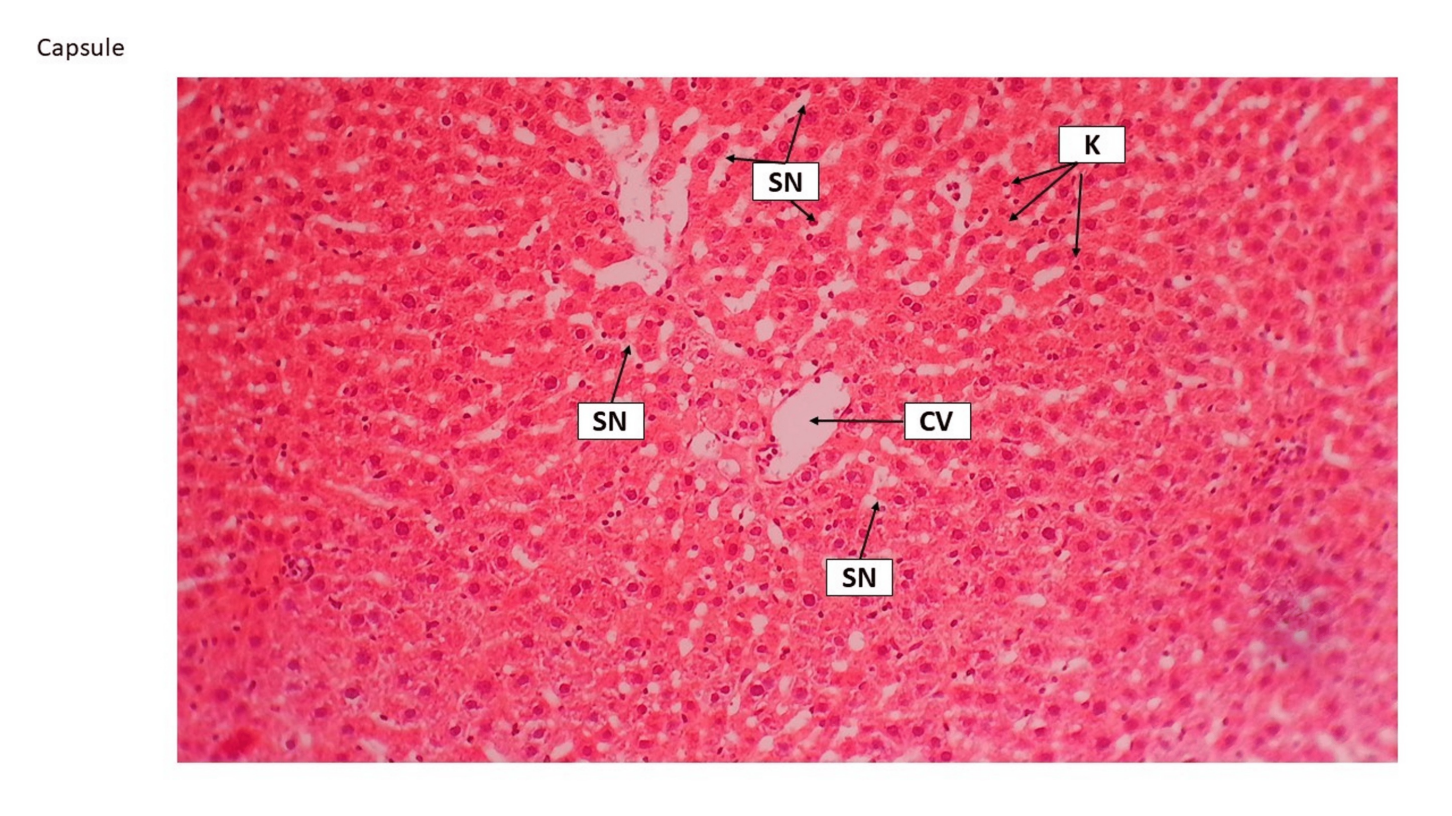
Slimming pill tea liver tissue group The slimming pill group revealed preserved histological features of the liver, including the central vein (CV), sinusoids (SN), and Kupffer cells (K). H&E staining 10×. H&E, hematoxylin and eosin

**Figure 8:**
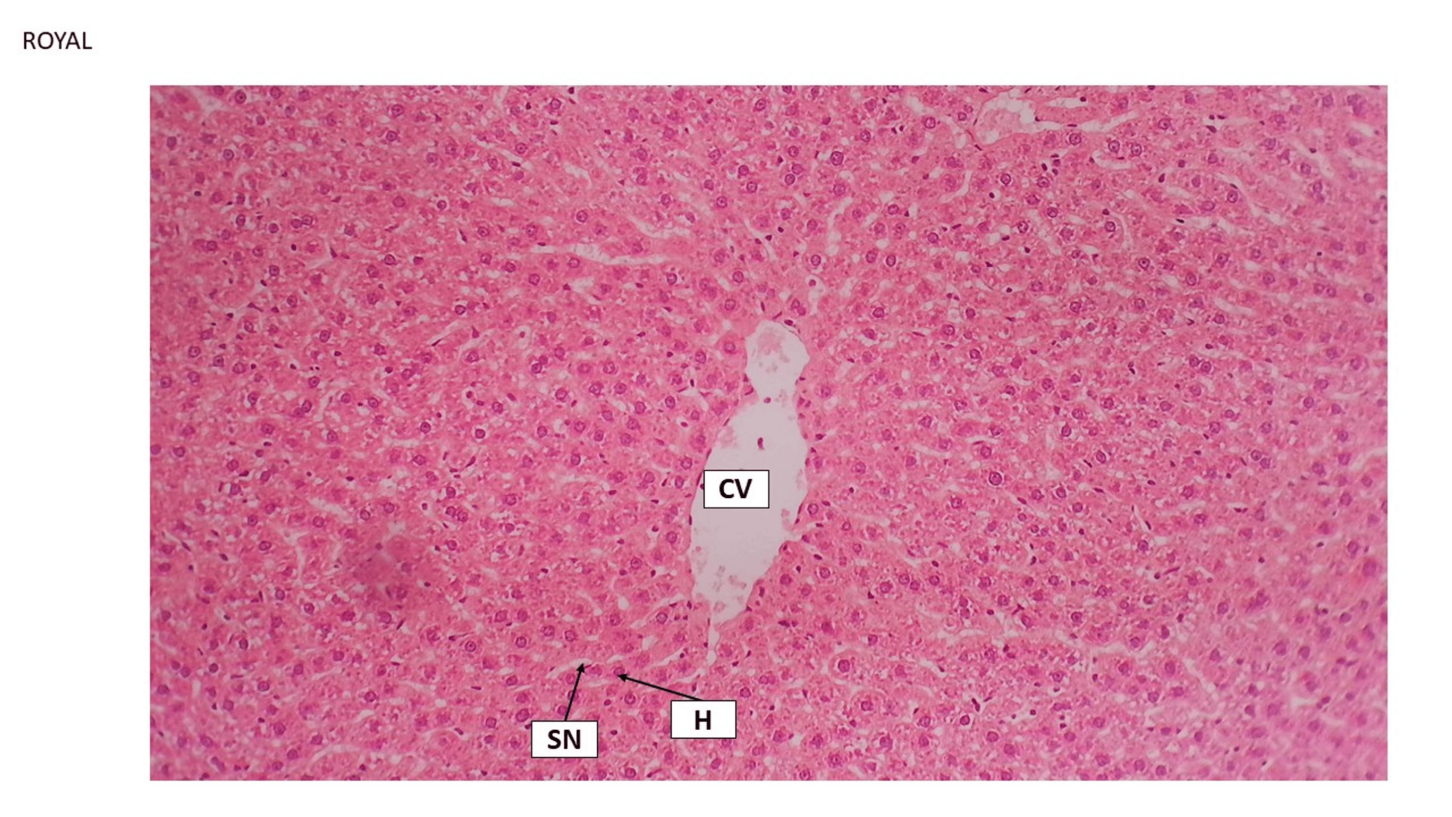
Shahana tea liver tissue group Shahana tea group throughout the section; normal histological features of the liver were preserved and revealed, including central vein (CV), sinusoids (SN) surrounded by hepatocytes (H) without any pathological changes. H&E staining 10×. H&E, hematoxylin and eosin

**Figure 9:**
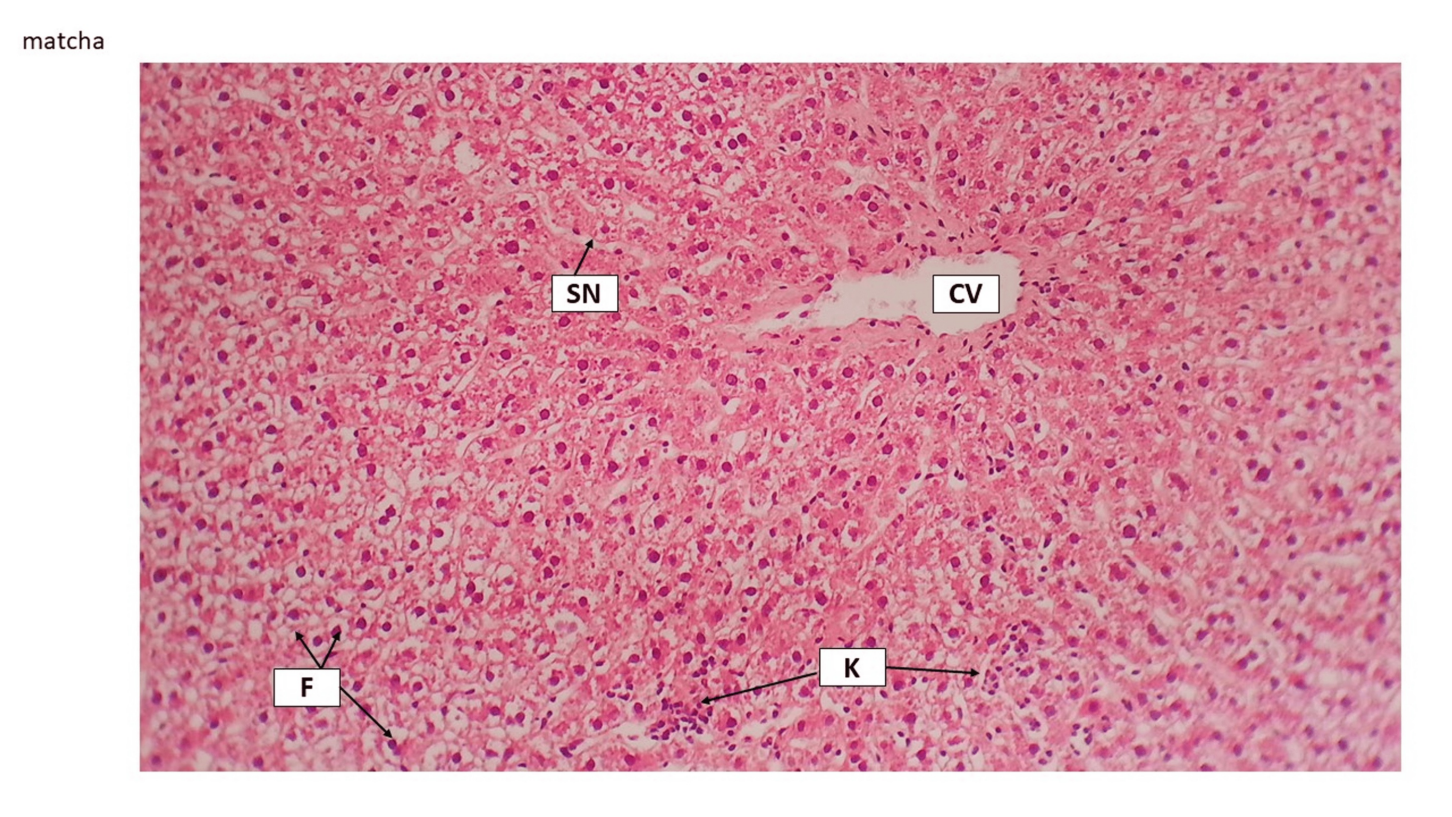
Japanese powder tea liver tissue group Japanese powder tea group revealed central vein (CV) and narrowed sinusoids (SN), hepatocytes showed fatty change (F), and there are foci of Kupffer cell hyperplasia (K). H&E staining 10×. H&E, hematoxylin and eosin

This finding has been explored by other studies, which have indicated that HFD and cholesterol cause fibrosis through various methods such as producing cytokines by Kupffer cells, inducing inflammation, apoptosis, and oxidative stress by hepatocytes, synthesis of collagen by satellite cells, and activating the toll-like receptor (TLR9)/inflammatory pathway by liver sinusoidal (SN) endothelial cells [6]. Slimming pills and Shahana tea could preserve the normal histological features of the liver. For instance, the central vein (CV), sinusoids, and Kupffer cells remained significantly unchanged. While Japanese powder tea did not indicate the pathological effect, the CV and sinusoids were narrowed, and hepatocytes showed filling with fat. Hyperplasia was identified in the foci of Kuppler cells (Figure 7, Figure 8, and Figure 9).

L-carnitine is the main component of slimming pills; it can regenerate injured hepatic cells by improving lipid metabolism. L-carnitine can inhibit inflammation and reduce lipid droplets to hepatic cells by increasing lipid metabolism; therefore, it can regenerate hepatic tissue [19,20]. L-carnitine can regenerate both parenchymal cells and non-parenchymal cells in the liver; it can preserve liver weight to control or normal level [20,21]. Yerba mate is another main component of slimming pill; the supplementing of yerba mate can reduce hepatic glutathione (GSH)/glutathione disulfide (GSSG), which promotes hepatoprotective and enhance the liver buffer for the oxidative stress [22].

Contrasting to Japanese powder tea, Shahana tea had a significant effect on liver tissue preservation rather than improving serum lipid profile. Shahana tea has *Cassia angustifolia* and phenolic components, which can protect hepatic histoarchitecture from ROS accumulation, and it can reduce the lipid peroxidation in the liver, glutathione (GSH), and malondialdehyde [18,23]. Compared to Japanese powder tea, Shahana tea has many hepatoprotective components that can ameliorate the toxicity risk on the liver in long-term therapy. *Ocimum basilicum* is another main component of Shahana tea, which can exhibit anti-inflammatory and anti-oxidative activity, prevent DNA damage and cell cycle arrest, and decrease levels of malondialdehyde [24,25].

The limitation of this study is related to the multitude of components of the slimming pill and Shahana tea. Each component and gradient in these teas, such as L-carnitine, Garcinia cambogia, vitamin B, *Cassia angustifolia*, *Juniperus communis*, *Ocimum basilicum*, sage leaves, mint leaves, and *Ceratonia*, could have its own particular mechanism on lipid metabolism and liver pathophysiology. Therefore, their effect requires to be measured independently. In this study, liver health has been measured through only pathological images; however, to measure the effect of those herbal teas on liver function, enzymes such as alanine transaminase (ALT), aspartate transaminase (AST), and alkaline phosphatase (ALP) should be measured. A low sample size, 20 rats or four rats in each group, was considered as another limitation of this study. The mean of lipid profile indicators, such as HDL, LDL, TG, and TC, could be more representative in a high sample size study.

## Conclusions

Slimming pills could significantly reduce the TC, TG, HDL, and LDL levels compared to Japanese powder tea and Shahana tea in HFD rats. Compared to the Shahana tea, Japanese powder tea has a significant improvement in LDL only, not other lipid profiles. Slimming pills and Shahana tea could preserve the normal histological features of the liver; the CV and SN Kupffer cells remained significantly unchanged in these teas. Herbal tea, due to its gradient, such as L-carnitine in slimming pill and *Cassia angustifolia* in Shahana tea, have significant positive effects on lipid metabolism regulation, protecting the body from dyslipidemia, and preserving the liver from injury, accumulation of fat, in HFD rats.
